# Phantom limb telescoping in individuals with limb loss: links to anxiety, depression, and pain-related measures

**DOI:** 10.3389/fpain.2026.1755884

**Published:** 2026-02-13

**Authors:** Andrea Aternali, Heather Lumsden-Ruegg, Lora Appel, Sander L. Hitzig, Amanda L. Mayo, Joel Katz

**Affiliations:** 1 Department of Psychology, York University, Toronto, ON, Canada; 2 School of Health Policy & Management, York University, Toronto, ON, Canada; 3 KITE, University Health Network, Toronto, ON, Canada; 4 GIM, Michael Garron Hospital, Toronto, ON, Canada; 5 St. John’s Rehab Research Program, Sunnybrook Research Institute, Sunnybrook Health Sciences Centre, Toronto, ON, Canada; 6 Department of Occupational Science and Occupational Therapy, Temerty Faculty of Medicine, University of Toronto, Toronto, ON, Canada; 7 Rehabilitation Sciences Institute, Temerty Faculty of Medicine, University of Toronto, Toronto, ON, Canada; 8 Dalla Lana School of Public Health, University of Toronto, Toronto, ON, Canada; 9 Department of Medicine, Temerty Faculty of Medicine, University of Toronto, Toronto, ON, Canada

**Keywords:** limb loss, phantom limb pain, post-amputation pain, residual limb pain, telescoping

## Abstract

Phantom limb pain (PLP) and residual limb pain (RLP) have been widely studied following limb loss; however, the role of telescoping, the perceived shortening of the phantom limb, remains poorly understood in pain and psychosocial outcomes. Using a cross-sectional observational design, this study examined whether PLP and RLP intensity, pain interference, and psychosocial functioning differ between individuals who report telescoping and those who do not. Fifty-one adults with limb loss (mean age = 49.5 years, SD = 15.4) completed measures of PLP and RLP intensity (0–10 numeric rating scale), telescoping (presence and percent), pain interference (Brief Pain Inventory–Short Form), pain catastrophizing (Pain Catastrophizing Scale–4), neuropathic pain (ID Pain Questionnaire), pain acceptance (Chronic Pain Acceptance Questionnaire–8), anxiety and depression symptoms (Patient Health Questionnaire–4), optimism (Life Orientation Test–Revised), and resilience (Connor–Davidson Resilience Scale–2). Twenty-three participants (45.1%) reported telescoping, while 28 (54.9%) did not. Telescoping was more common among younger participants and those with upper-limb loss, particularly right-sided below-elbow loss (all *p*s < .05). No significant between-group differences were observed for PLP intensity, RLP intensity, or pain interference (all *p*s > .05). However, greater percent telescoping was associated with lower pain interference (*r* = –.43, *p* = .040) and lower PLP intensity (*r* = –.49, *p* = .018). Participants reporting telescoping also endorsed higher symptoms of anxiety (*p* = .022) and depression (*p* = .029) relative to those with normal length phantoms. These findings suggest that telescoping may reflect distress linked to symptom monitoring and potentially adaptive cortical reorganization associated with reduced PLP.

## 1 Introduction

Limb loss is a major life-altering event that is frequently accompanied by acute and chronic pain conditions (1, 2). Among these, two of the most common and distressing consequences reported by individuals with limb loss are phantom limb pain (PLP), referring to painful sensations perceived as originating from the amputated or missing limb, and residual limb pain (RLP), pain localized to the remaining portion of the limb (3, 4). The majority of individuals with an amputation will experience one or both types of pain, often at clinically significant levels and for years after their amputation (1). Pain interference, defined as the extent to which pain disrupts daily activities, social roles, sleep, and mood, is also elevated following limb loss (5). Both PLP and RLP have been associated with greater interference in everyday functioning (6, 7). Importantly, amputation has been identified as the surgical procedure with the highest incidence of chronic post-surgical pain in comparison to other surgeries including thoracotomy, mastectomy, inguinal hernia, caesarian section, etc. (8). This underscores the need for continued investigation into the mechanisms and predictors of pain following limb loss (9).

Some individuals with limb loss also report other phantom limb phenomena, including telescoping, a phenomenon in which the phantom limb is perceived to progressively shorten and even retract completely into the residual limb. It was first described by Guéniot (10) and has since been examined in greater detail by others (11, 12). Telescoping is estimated to occur in approximately 22% of individuals with limb loss (13) but estimates vary considerably, ranging from 14%–32% in large samples (14–16).

The extent to which telescoping is linked to variations in pain intensity or psychosocial outcomes is not well established (17). Several studies have examined the relationship between telescoping and PLP, but findings have been inconsistent, with some studies reporting positive associations with PLP intensity, others reporting negative associations, and still others finding no significant relationship. Larger-sample size investigations have reported that telescoping may be linked to the presence of PLP (13, 18), whereas several smaller studies (19, 20) and case reports (21, 22) described the absence of PLP to be associated with telescoping. Other research, including both longitudinal (23) and experimental studies (24), have indicated that telescoping may coincide with lower levels of PLP intensity or even remission of pain. Taken together, these mixed findings highlight the uncertainty regarding whether telescoping is a risk factor for PLP, a protective mechanism against PLP, or a causally-unrelated correlate of PLP. Clarifying the nature of this relationship is important, as it may determine whether telescoping is a process that facilitates adaptation after amputation or one that hinders it. Furthermore, if telescoping can be shaped through targeted interventions, it raises important questions about whether therapeutic strategies, such as immersive virtual reality experiences, could be designed to encourage adaptive forms of telescoping and support post-amputation adjustment.

Psychosocial factors such as pain catastrophizing, characterized by heightened negative thinking and worry about pain, as well as anxiety and depression have been consistently associated with poorer outcomes in the broader chronic pain literature, including conditions such as low back pain, fibromyalgia, and arthritis (25). In these conditions, the above psychosocial factors have been associated with increased pain intensity, greater disability, and diminished quality of life (26–32). Similarly, anxiety and depression have been linked to poor adjustment, greater pain, and less successful rehabilitation outcomes after limb loss (33, 34). These negative affective and cognitive processes may exacerbate pain perception and interfere with adaptive coping, highlighting the importance of examining psychological contributors to post-amputation pain experiences. In contrast, positive constructs such as optimism, resilience, and acceptance may serve as protective factors, potentially buffering individuals against pain-related distress and facilitating more effective coping strategies (35–37). Together, these constructs allow for a comprehensive investigation of both vulnerability and resilience factors in the context of chronic post-amputation pain.

While PLP and RLP have been extensively studied, the role of telescoping remains less understood in shaping these pain experiences and associated psychosocial outcomes. Very few investigations have directly compared individuals who report telescoping with those who do not, particularly in relation to pain intensity and psychosocial dimensions such as pain catastrophizing, anxiety, and resilience. This lack of direct comparison represents a critical gap in the literature. As such, the present study aims to examine how telescoping is associated with psychosocial aspects of post-amputation pain. Specifically, the goal was to establish whether pain intensity and interference, as well as psychosocial functioning, including pain catastrophizing, anxiety, depression, optimism, and resilience, differ between those who endorse telescoping and those who do not. Given the current lack of evidence regarding whether telescoping is a risk factor, a protective factor, or is causally unrelated to PLP, we did not make specific hypotheses about the direction of the relationships. Instead, our primary objective was to examine whether individuals with and without telescoping differ systematically in pain-related factors and psychosocial variables.

## 2 Methods

### 2.1 Recruitment

Eligible participants were adults over the age of 18 years old, fluent in English, who had undergone one or more upper and/or lower limb amputations of any etiology at least three months prior to recruitment. Individuals were excluded if they had significant cognitive impairment, a diagnosis of severe psychopathology (e.g., active psychotic disorder, dementia), or significant issues in visual impairment that would interfere with study participation.

The study team implemented multiple recruitment strategies from June 1, 2019 to December 16, 2024 to reach eligible individuals. (1) Treating clinicians at participating hospital sites in Toronto, Ontario introduced the study to eligible patients and provided preliminary information. (2) Flyers outlining the study purpose, eligibility criteria, compensation, and research team contact information were posted in hospital areas serving individuals with limb loss. (3) The same flyer was disseminated electronically via websites, forums, and social media platforms to increase study visibility. (4) Finally, individuals who had previously consented to be contacted for research participation through the St. John's Rehab Research Database received an email invitation containing a brief study description and eligibility criteria.

Interested individuals were asked to contact the research team by phone or email. Upon expressing interest, potential participants received a unique, anonymous study ID and a secure link to the online consent form. Those who provided consent were directed to online questionnaires and a web-based app, which in total required approximately 45 min to complete. Individuals who declined consent were thanked for their time and exited from the study platform.

The online consent form also provided participants the option to take part in a qualitative interview regarding their experiences of phantom limb pain and telescoping. Only participants who both expressed interest and endorsed telescoping on the online measures were invited to complete the optional interview. The results of the qualitative interview study will be published in a separate article. Ethics for this study was provided through the Research Ethics Boards at the Sunnybrook Health Sciences Centre (REB# 3071) and York University (HPRC #e2019-138).

### 2.2 Questionnaires

Pain interference was assessed using the Brief Pain Inventory—Short Form (BPI-SF) (38), which evaluates the impact of pain on seven domains, including general activity, mood, work, sleep, and enjoyment of life. Each item is rated on a scale from 0 (“does not interfere”) to 10 (“completely interferes”), and the average score across items provides a reliable and valid measure of pain interference across a range of conditions, including phantom limb pain (34).

Neuropathic pain was screened for using the 6-item ID Pain Questionnaire (IDPQ), a self-administered tool with “yes” or “no” response options (39). The items assess neuropathic pain characteristics such as burning, numbness, and electric-like sensations. Scores range from −1 to 5, with higher scores reflecting a greater likelihood of neuropathic pain; scores between 3 and 5 are associated with an estimated 69% probability of neuropathic pain (39). The IDPQ has been shown to effectively identify pain with a neuropathic component (39).

Pain-related catastrophic thinking was measured with the four-item version of the Pain Catastrophizing Scale (PCS-4), which uses a 5-point Likert scale from 0 (“not at all”) to 4 (“all the time”) (40). Total scores range from 0 to 16, with higher scores indicating greater catastrophic thinking about pain. The PCS-4 demonstrates strong internal consistency and correlates highly with the full PCS (40, 41).

Depression and anxiety symptoms were assessed using the Patient Health Questionnaire-4 (PHQ-4) (42). Scores on the first two items contribute to the anxiety symptoms subscale, and scores on the last two items contribute to the depression symptoms subscale. Total scores range from 0 to 12, with categories of normal (0–2), mild (3–4), moderate (6–8), and severe (9–12). Subscale scores ≥3 are considered indicative of clinically relevant symptoms. The PHQ-4 is a brief measure of these affective symptoms that has previously demonstrated good reliability and validity (43).

Dispositional optimism was measured using the 10-item Life Orientation Test—Revised (LOT-R) (44). Participants rate statements about positive and negative expectations on a 5-point scale from 0 (“strongly disagree”) to 4 (“strongly agree”), with four items serving as fillers. Total scores range from 0 to 24, with higher scores indicating greater optimism. The LOT-R includes positively and negatively worded items and shows good reliability and validity in both general and chronic pain populations (45).

Resilience, defined as the ability to recover and adapt to change, was assessed using the 2-item Connor-Davidson Resilience Scale (CD-RISC2) (46). Items are rated from 0 (“not at all true”) to 4 (“true nearly all the time”), producing a total score from 0 to 8, with higher scores reflecting greater resilience. The CD-RISC2 demonstrates strong internal consistency, convergent and divergent validity, and test-retest reliability, with a mean score of approximately 6.91 in North American populations (46).

Acceptance of chronic pain was evaluated with the 8-item Chronic Pain Acceptance Questionnaire (CPAQ-8) (47). Participants rate each item from 0 (“never true”) to 6 (“always true”). The measure includes subscales of pain willingness and activity engagement, which combine to produce a total score from 0 to 48, where higher scores indicate greater pain acceptance. The measure has demonstrated strong validity and psychometric reliability in rehabilitation contexts (48).

### 2.3 Telescoping app

**Figure 1:**
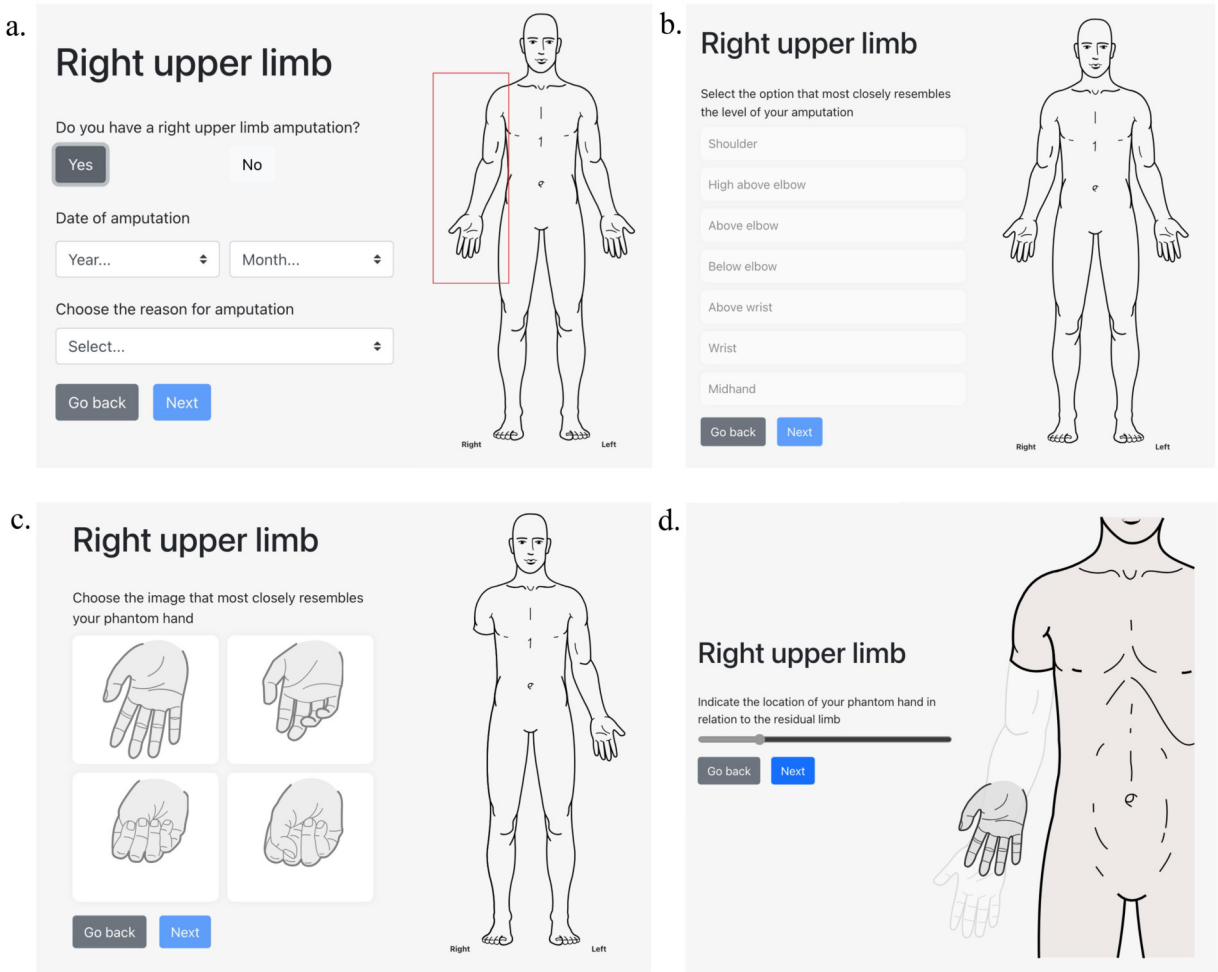
Example of the telescoping app interface for a right upper limb. Once the individual selects a limb (in this case, the right upper limb), they are asked to input the date and reason for their amputation **(a)**. After this has been entered, they are asked to identify the level of their amputation **(b)**. Next, they may choose the image which best represents their phantom hand **(c)** or foot in the case of a lower limb amputation. Lastly, they are asked to use a slider to position their hand (or foot) where they usually feel it in relation to their residual limb **(d)**.

Participants used a web-based/mobile application (https://demo.phantomlimbs.ca/) developed by our team to assess the presence and extent of phantom limb telescoping and related factors. Users enter basic demographic and clinical information (e.g., sex, month and year of birth, month and year of amputation), indicate the affected side, limb and level of amputation, select the posture of their phantom limb hand or foot from among four common exemplars, and visually quantify the degree of telescoping using a slider that moves the phantom limb from normal length to fully telescoped within the residual limb (49). Figure 1 depicts the participant's view while using the app, illustrated with a right upper limb example. The app was designed to be quick to complete while also capturing amputation-related information not captured by other similar tools (50). Participants were asked to rate the intensity of the PLP and RLP they experienced several times a week or more over the past 3 months using a 0–10 numeric rating scale slider, where 0 refers to “no pain” and 10 to “worst possible pain”. The app calculates the percentage of telescoping relative to the length of a normal limb (0%) to fully telescoped into the residual limb (100%). The application was developed to provide a consistent, precise, and systematic method for documenting telescoping and investigating its association with post-amputation pain.

### 2.4 Analyses

Descriptive statistics were used to summarize participant demographic and clinical characteristics. Means and standard deviations were calculated for continuous variables, and frequencies and percentages were calculated for categorical variables.

Between-group comparisons were conducted to examine differences between participants who reported telescoping and those who reported normal phantom limb length. Independent-samples *t* tests were used to compare continuous demographic, clinical, and psychosocial variables (e.g., age, pain intensity ratings, pain interference, catastrophizing, optimism, resilience, depression, anxiety, and pain acceptance). For all *t* tests, Levene's test for equality of variances was conducted; results are reported using the equal variances assumed statistic when Levene's test was non-significant and the equal variances not assumed statistic when Levene's test was significant. Chi-square tests of independence were conducted to compare categorical variables (e.g., sex, ethnicity, marital status, education, living situation, amputation etiology, and amputation level). In addition, within the subgroup of participants who endorsed telescoping, Pearson correlations were conducted to examine associations between the percentage of telescoping reported and continuous pain (intensity, interference) and psychosocial variables (e.g., age, catastrophizing, resilience, depression, anxiety, pain acceptance, etc.).

Analyses of amputation type (upper/lower limb), side (left/right), and level (above/below elbow/knee) were performed at the limb level rather than the individual level, given that two participants had multiple amputations.

When chi-square tests indicated significant group differences, *post hoc* comparisons were performed with Bonferroni correction to control for multiple comparisons. All tests were two-tailed with a significance threshold of *p* < .05. Missing data were handled by listwise deletion, such that participants who did not complete an entire questionnaire, such as the telescoping app, were not included in the final analytic sample. For participants who completed a questionnaire but had one missing item, the missing value was replaced with the individual's average response on that questionnaire (51). All analyses were conducted using *IBM SPSS Statistics, Version 31* (IBM Corp., Armonk, NY).

## 3 Results

### 3.1 Participant characteristics

A total of 86 individuals expressed interest in participating in the study. Eighteen were excluded because their email addresses appeared suspicious (e.g., random strings of letters followed by numbers followed by an email service address) and they declined to provide relevant information necessary for participation (e.g., responses to inclusion/exclusion criteria, full name, or contact information). Eleven individuals provided their contact information but did not respond to subsequent outreach from the research assistant, who had attempted to provide them with additional study details. Six individuals provided consent but did not complete all online questionnaires.

Fifty-one participants comprised the final sample size for data analysis. Thirty-three individuals (64.7%) identified as male and 18 (35.3%) identified as female. Participants had a mean age of 49.5 years (SD = 15.4, range = 18–79). Most participants identified as White (76.5%), were married or in a domestic partnership (52.9%), and lived with a spouse or significant other (62.7%). The majority had reported obtaining either an associate degree or college diploma/certificate (33.3%) or bachelor's degree (29.4%) as their highest level of education.

Nearly all participants (96.1%) had undergone amputation of one limb, with the exception of two participants (i.e., one who lost both lower extremities and another who lost all four limbs). Accordingly, limb-level analyses included 55 limbs rather than 51 participants. Of the 55 limbs, 11 were upper limbs and 44 were lower limbs. The most common cause of amputation was trauma (49.0%), followed by diabetes and/or vascular disease (21.6%), malignancy (5.9%), and non-diabetic/vascular-related infection (2.0%). The remaining 21.5% of participants reported another cause of amputation. Time since amputation averaged 5.7 years (SD = 7.3, range = 3.8 months to 38.1 years). A majority of participants (70.6%) reported experiencing ongoing pain. Ratings of PLP and RLP intensity were 3.6 (SD = 3.1) and 4.3 (SD = 3.0), respectively, on a 0–10 numerical rating scale. At the participant level, 23 individuals (45.1%) endorsed telescoping and 28 (54.9%) did not. At the limb level, 24 of 55 limbs (43.6%) were reported to be telescoped to some degree, and 31 (56.4%) did not. Among cases endorsing telescoping, the mean percent telescoping was 23.5% (SD = 31.5; range = 0–100).

### 3.2 Group comparisons

Table 1 presents the demographic and clinical characteristics of participants who endorsed telescoping and those who did not. Chi-square analyses revealed that the two groups did not significantly differ in sex distribution, highest education completed, ethnicity, marital status, living situation, presence of ongoing pain problems, amputation etiology or side of amputation (e.g., left or right). Independent-samples *t*-tests revealed no significant differences between the two groups on the number of ongoing pain problems, number of limbs amputated, or time since amputation. Individuals who reported telescoping were significantly younger (M = 42.2 years, SD = 15.5) than those who did not (M = 55.5 years, SD = 12.7), *t*(49) = 3.37, *p* = .001.

**Table 1:** Demographic and clinical characteristics for individuals with vs. without telescoping.

Variable	Telescoping (*n* = 23)	Normal length (*n* = 28)	*P*
Sex, *n* (%)			.066
Male	18 (78.3)	15 (53.6)	
Female	5 (21.7)	13 (46.4)	
Age (y), mean, SD (range)	42.2, 15.5 (18–72)	55.5, 12.7 (28–79)	.001*
Highest education completed, *n* (%)			.498
High school	1 (4.3)	2 (7.1)	
High school/GED	3 (13.1)	4 (14.3)	
College/Associate degree	6 (26.1)	11 (39.3)	
Bachelor's degree	10 (43.5)	5 (17.9)	
Graduate degree	1 (4.3)	3 (10.7)	
Professional degree	2 (8.7)	3 (10.7)	
Ethnicity, *n* (%)			.117
White	16 (69.6)	23 (82.1)	
African Canadian/Caribbean	4 (17.5)	0 (0.0)	
East Asian	1 (4.3)	0 (0.0)	
Hispanic/Latino/a	0 (0.0)	1 (3.7)	
Middle Eastern/North African	1 (4.3)	0 (0.0)	
South Asian	1 (4.3)	2 (7.1)	
Other	0 (0.0)	2 (7.1)	
Marital status, *n* (%)			.435
Single/Never married	6 (26.2)	6 (21.4)	
In a relationship	5 (21.7)	2 (7.1)	
Married/Domestic partnership	9 (39.2)	18 (64.3)	
Widowed	1 (4.3)	1 (3.6)	
Divorced	1 (4.3)	1 (3.6)	
Separated	1 (4.3)	0 (0.0)	
Living situation, *n* (%)			.225
Living alone	2 (8.7)	3 (10.7)	
With roommate (s)	1 (4.3)	2 (7.1)	
With relative(s)	8 (34.8)	3 (10.7)	
With spouse/significant other	12 (52.2)	20 (71.5)	
Amputation etiology, *n* (%)			.362
Accident/Trauma	14 (60.9)	11 (39.3)	
Cancer/Tumor	1 (4.3)	2 (7.1)	
Diabetes/Vascular disease	3 (13.1)	8 (28.6)	
Non-diabetic vascular infection	1 (4.3)	0 (0.0)	
Other	4 (17.4)	7 (25.0)	
Any ongoing pain problems, *n* (%)			.884
Yes	16 (69.6)	20 (71.4)	
No	7 (30.4)	8 (28.6)	
Number of ongoing pain problems, mean, SD (range)	1.3, 1.4 (0–5)	1.1, 1.0 (0–3)	.482
Number of limbs amputated, mean, SD (range)	1.1, 0.6 (1–4)	1.0, 0.2 (1–2)	.450
Type of amputation^a^, *n* (%)			.030*
Upper limb	8 (33.3)	3 (9.7)	
Lower limb	16 (66.7)	28 (90.3)	
Side of amputation^a^, *n* (%)			.739
Right	15 (62.5)	18 (58.1)	
Left	9 (37.5)	13 (41.9)	
Side and type of amputation^a^, *n* (%)			.023*
Right upper limb	5 (20.8)	0 (0.0)	
Right lower limb	9 (37.5)	16 (51.6)	
Left upper limb	3 (12.5)	1 (3.2)	
Left lower limb	7 (29.2)	14 (45.2)	
Level of amputation^a^, *n* (%)			.024*
Below-elbow	4 (16.7)	0 (0.0)	
Above-elbow	4 (16.7)	1 (3.2)	
Below-knee	12 (49.9)	24 (77.4)	
Above-knee	4 (16.7)	6 (19.4)	
Time since amputation (y), mean, SD (range)	5.5, 6.4 (0.3–26.8)	5.9, 8.0 (0.3–38.1)	.838

y; years, SD; standard deviation.

a Variables assessed at the limb level (*n* = 55, where 24 limbs were reported to experience telescoping and 31 were normal length phantoms).

* *p* < .05.

Analyses did show a significant difference between groups for type of amputation (e.g., upper or lower), *χ*²(1, *N* = 55) = 4.73, *p* = .030. Specifically, telescoping was reported in 33.3% of individuals with an upper limb amputation, compared to 9.7% of individuals with a lower limb amputation. Similarly, there was a significantly greater proportion of individuals with lower limb amputations who did not report telescoping (90.3%) than those who reported telescoping (66.7%). There were also significant group differences when type of amputation (e.g., upper or lower) and side of amputation (e.g., left or right) were evaluated together, *χ*²(1, *N* = 55) = 9.56, *p* = .023. Bonferroni-adjusted *post hoc* analyses revealed a significantly greater proportion of individuals with a right upper limb amputation in the telescoping group than the normal length phantom group. All other group comparisons were not significant. Further, there were significant differences between groups for level of amputation (e.g., above/below elbow/knee), *χ*²(1, *N* = 55) = 9.46, *p* = .024. Bonferroni-adjusted *post hoc* analyses revealed a significantly greater proportion of individuals with a below-elbow amputation endorsed telescoping than those who did not report telescoping. Fewer individuals with a below-knee amputation who endorsed telescoping than those who did not report telescoping. All other group comparisons were not significant.

Pain (intensity and interference) ratings and psychosocial variables were compared between individuals with telescoping and those without (see Table 2). Independent samples *t* tests did not reveal significant group differences in RLP intensity, *t*(49) = −0.553, *p* = .583, or PLP intensity, *t*(49) = 0.245, *p* = .808. Similarly, there were no group differences for pain interference (BPI-SF), *t*(49) = −.288, *p* = .775, pain catastrophizing (PCS-4), *t*(49) = −.724, *p* = .473, optimism (LOT-R), *t*(49) = 0.973, *p* = .335, or resilience (CD-RISC 2), *t*(49) = 0.567, *p* = .573. Conversely, differences were observed for anxiety and depression, whereby participants with telescoping reported higher anxiety symptoms (PHQ-4), *t*(49) = −2.385, *p* = .022, as well as higher symptoms of depression (PHQ-4), *t*(49) = −2.252, *p* = .029.

**Table 2:** Pain ratings and psychosocial variables for individuals with vs. without telescoping.

Variable	Telescoping (*n* = 23)	Normal length (*n* = 28)	*P*
Residual limb pain, mean, SD	3.9, 3.0	3.4, 3.3	.583
Phantom limb pain, mean, SD	4.2, 2.8	4.4, 3.3	.808
BPI-SF pain interference, mean, SD	3.7, 2.6	3.5, 2.8	.775
PCS-4, mean, SD	7.6, 5.4	6.6, 3.9	.473
PHQ-4, mean, SD			
Anxiety	2.7, 2.2	1.3, 1.7	.022*
Depression	2.5, 2.2	1.3, 1.6	.029*
LOT-R, mean, SD	14.7, 5.2	16.0, 4.4	.335
CD-RISC2	6.3, 1.5	6.6, 1.3	.573
CPAQ-8^a^, mean, SD			
Willingness	13.1, 4.7	12.0, 3.5	.440
Activity engagement	16.5, 4.2	16.9, 4.2	.779
Total	29.6, 7.4	28.9, 4.7	.747
ID Pain^a^, mean, SD	2.9, 1.1	2.7, 1.4	.599

SD, standard deviation; BPI-SF, brief pain inventory—short form; PCS-4, pain catastrophizing scale; PHQ-4, patient health questionnaire-4; LOT-R, life orientation test—revised; CD-RISC2, connor-davidson resilience scale; CPAQ-8, chronic pain acceptance questionnaire.

a Variables assessed using those who endorsed ongoing pain problems (*n* = 36, where 16 reported telescoping and 20 had normal length phantoms).

* *p* < .05.

Among the 36 participants who endorsed ongoing pain problems, pain acceptance scores (CPAQ) were compared between those with telescoping (*n* = 16) and those with normal length perception (*n* = 20), as shown in Table 2. Independent samples *t* tests did not reveal group differences for the willingness, *t*(34) = −.781, *p* = .440, or activity engagement, *t*(34) = .283, *p* = .779, subscales. Similarly, no differences were detected between groups on the total CPAQ score, *t*(34) = −.326, *p* = .747. No other group differences on other measures were detected.

### 3.3 Correlational analyses

A greater degree of telescoping was associated with lower pain interference (BPI-SF), *r*(21) = –.431, 95% CI [–.716, –.022], *p* = .040, and lower PLP intensity, *r*(21) = –.490, 95% CI [–.751, –.098], *p* = .018. No other significant correlations were observed with any other variable (all *ps* > .05; see Table 3).

**Table 3:** Correlations between precent of telescoping and psychosocial variables among participants who endorsed telescoping.

Variable	Pearson correlation	*P*
Age	−.316	.142
Time since amputation	.347	.104
RLP (NRS)	−.298	.168
PLP (NRS)	−.490	.018*
BPI-SF	−.431	.040*
PCS-4	−.134	.542
PHQ-4		
Anxiety	−.089	.688
Depression	−.157	.475
LOT-R	−.173	.429
CD-RISC2	.055	.801
CPAQ-8^a^		
Willingness	−.178	.510
Activity engagement	−.405	.119
Total	−.342	.195
ID Pain^a^	.092	.736

RLP, residual limb pain; PLP, phantom limb pain; BPI-SF, brief pain inventory—short form; PCS-4, pain catastrophizing scale; PHQ-4, patient health questionnaire-4; LOT-R, life orientation test—revised; CD-RISC2, connor-davidson resilience scale; CPAQ-8, chronic pain acceptance questionnaire.

a Variables assessed using those who endorsed ongoing pain problems (*n* = 16).

* *p* < .05.

## 4 Discussion

This study examined demographic, clinical, and psychosocial differences between individuals who reported telescoping of their phantom limb and those who did not, with the aim of clarifying the relationship between telescoping and phantom limb sensations, including pain. In this sample, 23 individuals (45.1%) reported experiencing telescoping of the phantom limb, while 28 (54.9%) did not. This rate is markedly higher than the 14%–33% estimate reported in large-scale studies (13–16). There are several plausible reasons for this discrepancy. First, the sample size is relatively small, which increases the likelihood of sampling variation and possibly overestimation of prevalence compared to those large-scale investigations. Second, the composition of our sample may differ in keyways. Most notably, a large proportion of participants had a traumatic amputation etiology, which prior work has suggested is associated with higher rates of phantom phenomena such as telescoping (17). Thus, the elevated rate of telescoping in our cohort may reflect greater risk factors (trauma etiology) in the sample, rather than indicating a fundamentally higher population rate. Finally, other sample-specific factors (e.g., time since amputation, level of amputation, prosthesis use, and pre-amputation pain) may also play a role in the difference. Thus, while the higher observed prevalence of telescoping highlights a meaningful occurrence in this cohort, caution is warranted in generalizing this rate to the broader population of individuals with limb loss. Future studies with larger, more heterogenous samples will help clarify the true prevalence of the telescoping phenomenon.

The findings of this study provide greater insights on the telescoping phenomenon by highlighting factors related to age, amputation location, and psycho-social profiles. For instance, those who reported telescoping tended to be younger than those who did not. This may reflect greater neuroplasticity earlier in adulthood, which could increase susceptibility to perceptual phenomena like telescoping (52). Younger individuals may also engage in higher activity levels and adopt prostheses more quickly, amplifying sensory input that may impact perceived limb length (53, 54). Nevertheless, evidence from prior studies suggests that reduced prosthesis use may be associated with shorter phantom limbs (55), while meaningful sensorimotor engagement through prosthesis use can lengthen previously shortened phantoms through use-dependent cortical reorganization (12, 56), highlighting the complexity of the relationship between sensory engagement and telescoping. In addition, the complicating role of vision when wearing a prosthesis may affect telescoping irrespective of use-dependent changes in brain reorganization since telescoped phantoms often lengthen to assume the proportions of the prosthesis as appreciated by sight (57). Younger individuals may also be more likely to notice and report phantom-related changes, whereas older adults may have more stable somatosensory maps and less pronounced distortions. In terms of other demographic or clinical characteristics, including sex, marital status, education, or time since amputation, no significant findings were detected, which may suggest that underlying neurocognitive or perceptual mechanisms related to body representation following limb loss play a larger role in telescoping.

Telescoping was more frequently reported among individuals with upper limb amputations compared to those with lower limb amputations, especially those who lost a right upper limb below the elbow. This finding suggests that limb type and dominance may influence the occurrence of this phenomenon. One possible explanation lies in differences in cortical representation, as upper limbs, and especially the dominant right hand, occupy greater sensory and motor cortical areas, which may make body image distortions such as telescoping more likely (58–60). Although hand dominance was not measured in this sample, population base rates suggest that most participants were likely right-handed. This pattern may align with some prior reports indicating higher prevalence of telescoping in upper than lower limb amputees (24, 61, 62). Future studies should include hand dominance as a variable to better account for this factor.

Although the present study did not find significant group differences in ratings of PLP intensity, RLP intensity, or pain interference, there was a negative association between PLP intensity and percent telescoping. One interpretation is that greater telescoping may reflect adaptive cortical reorganization that, for some individuals, co-occur with lower PLP intensity (11). Conversely, higher pain may disrupt embodiment and reduce telescoping. It is also plausible that effects are non-linear or moderated by factors such as age, level of amputation, or time since amputation, which could attenuate simple between-group contrasts but still yield within-group correlations (63). In line with this, recent work indicates that primary somatosensory hand representations can remain *stable* before and up to five years after upper-limb amputation (64). This individual variability in cortical reorganization may dilute differences between those who do and do not endorse telescoping, even as associations within a single group (e.g., those who endorse telescoping) still emerge. Ultimately, such factors would have to be investigated in larger samples.

In terms of psychological variables, participants who reported telescoping also endorsed significantly higher levels of anxiety and depression, which may reflect maladaptive coping or increased vigilance toward altered body perceptions such as a shorter than normal phantom limb. It is also possible that higher levels of anxiety or depression may contribute to increased attention to bodily sensations or perceptual distortions, which could influence the experience or reporting of telescoping. Further research is needed to clarify the direction of this relationship, though we hypothesize that the perceptual changes associated with telescoping may contribute to elevated anxiety and depressive symptoms rather than the reverse. The broader research evidence might therefore suggest that individuals who experience telescoping could be more vulnerable to increased pain. At the same time, between-group differences were not found between telescoping and PLP intensity, RLP intensity, or pain interreference. Further, within the telescoping subgroup, greater percentage of telescoping was associated with lower PLP intensity. Group differences also were not found in optimism or resilience, suggesting that telescoping is not uniformly associated with either protective or harmful psychosocial factors. Taken together, these findings suggest that telescoping should not be interpreted as a marker of greater pain intensity or interference. A dual-process interpretation may explain the results whereby: (a) the presence of telescoping may be distressing for some individuals, contributing to elevated symptoms of anxiety and depression via uncertainty and increased symptom monitoring, while (b) the degree of telescoping, once present, may be indicative of adaptive cortical reorganization that co-occurs with lower PLP intensity for a subset of participants. Clinically, this pattern points to a mixture of affective responses to an unusual sensation and, in some cases, potentially beneficial neuroplastic change.

These findings underscore the importance of incorporating psychosocial assessment into the care of individuals who report phantom limb telescoping, with particular attention to screening for anxiety and depression. The observed associations with anxiety and depression suggest that, for some individuals, telescoping may be accompanied by negative affective responses (e.g., uncertainty, heightened monitoring of boldly sensations, difficulty coping, etc.). Tailored interventions, such as acceptance and commitment therapy to address unhelpful coping strategies or cognitive-behavioral therapy to reduce symptoms of anxiety and depression, may help alleviate distress and support better psychological adjustment (65–67). Theoretically, the results contribute to ongoing debates regarding whether telescoping should be considered solely a perceptual phenomenon or one that is also intertwined with psychosocial outcomes.

## 5 Strengths and limitations

A notable strength of this study is its inclusion of both clinical and psychosocial variables, allowing for a more comprehensive understanding of the factors associated with telescoping beyond pain intensity alone. The use of standardized and validated measures for constructs such as pain, catastrophizing, anxiety, depression, and resilience further enhances the reliability and comparability of the findings. Likewise, the measure of telescoping used in the present study provides a more in-depth and precise way to quantify what has been a difficult phenomenon to assess. Despite these strengths, several limitations should be acknowledged. First, the relatively small final sample size (*N* = 51) reduced the statistical power to detect more subtle group differences and may limit the robustness of the observed effects. Second, because recruitment was conducted online, the sample may not be representative of the broader population of individuals with limb loss; in particular, the overrepresentation of telescoping and White participants raises concerns about generalizability across diverse demographic groups. Third, all measures relied on self-report, which introduces the possibility of recall bias and variability in how participants understood and reported telescoping. Fourth, participation bias may have occurred, as individuals experiencing higher levels of distress, depression, or limited social support may have been less likely to enroll, potentially influencing the generalizability of our findings. Finally, the cross-sectional design does not establish the temporal relationship between variables, preventing us from determining whether changes in telescoping preceded or followed the development of pain, and/or the psychosocial factors.

## 6 Conclusions and future directions

This study demonstrated that telescoping was more common among younger individuals with upper limb amputations, particularly on the right side, below the elbow, and was associated with higher levels of anxiety and depression. Percent telescoping was negatively associated with PLP intensity in correlational analyses. Nevertheless, no associations were present between telescoping and RLP intensity, pain interference, and most other psychosocial variables, including optimism and resilience. Taken together, these findings suggest that telescoping is not a stand-alone marker of overall pain burden and may reflect a mix of affective responses (e.g., anxiety/depression) and, for some individuals, adaptive reorganization. Clinically, the results highlight the value of routine screening for anxiety and depression, and they point to the potential utility of supportive interventions aimed at improving adjustment among individuals who report telescoping.

Future research should extend these findings by examining the longitudinal course of telescoping and its relationship with psychosocial adjustment over time. Prospective studies could help determine whether telescoping precedes or follows psychological distress, thereby clarifying its causal role. Neuroimaging approaches may also provide important insights into the cortical reorganization and sensorimotor mechanisms underlying telescoping. Intervention studies are needed to test whether strategies such as cognitive-behavioral therapy for catastrophizing or mindfulness-based approaches for anxiety can reduce distress linked to telescoping. Additionally, recruiting larger and more diverse samples will improve the generalizability of results, while incorporating rich qualitative approaches is essential to capture the lived experience of telescoping, deepen understanding of the phenomenon, and inform the design of meaningful and sustainable interventions.

## Data Availability

The raw data supporting the conclusions of this article will be made available by the authors, without undue reservation.
